# Return to Play After the Diagnosis of Reactive Arthritis in a Professional Football Player

**DOI:** 10.7759/cureus.41139

**Published:** 2023-06-29

**Authors:** Alexandre Fernandes, Pedro Cunha, Julio Pinto, Carlos Duarte, Alexandre Estaca, Tiago Pereira, Mónica Bettencourt, Miguel Reis e Silva, Susana Fernandes

**Affiliations:** 1 Physical Medicine and Rehabilitation, Hospital de Cascais, Lisbon, Lisbon, PRT; 2 Health and Performance Unit, Casa Pia Atlético Clube - Futebol Sduq, Lisbon, PRT; 3 Physiotherapy - Health and Performance Unit, Casa Pia Atlético Clube - Futebol Sduq, Lisbon, PRT; 4 Sports Medicine, Myalgia Clinic, Lisbon, PRT; 5 Rheumatology, Hospital dos Lusíadas, Lisboa, Lisbon, PRT

**Keywords:** professional sport, reactive arthritis, return-to-play, rehabilitation, elite football

## Abstract

In professional football, most of the injuries are traumatic; however, these athletes may suffer from rheumatologic diseases, that may present as sports-related injuries. Reactive arthritis (ReA) is classified as a sub-group of the spondyloarthritis family and is relatively rare. In this article, we highlight the successful return to play (RTP) process after the ReA diagnosis in an elite football player in the Portuguese first league. The athlete was able to RTP four months and one week after the diagnosis, had no ReA recurrence nor re-injury >8 months after RTP, and is playing at an elite level.

## Introduction

Football is the most popular sport worldwide [1]. Injuries are common in professional football and are the most common reason for player unavailability in training and matches, being shown that a high injury burden affects the team's performance negatively [2].

According to the Union of European Football Associations (UEFA) Elite Club Injury Study Report 2019/20, most of the injuries sustained were traumatic, being 67% of total injuries [3,4]. There is a temptation to attribute a sports-related diagnosis to every patient who presents with a joint complaint [5]. However, these athletes may suffer from systemic rheumatologic diseases, that may initially mimic sports-related injuries [5,6]. These conditions often affect the musculoskeletal system and may mimic traumatic and overuse injuries initially [5].

Reactive arthritis (ReA) is a rheumatologic condition classified as a sub-group of the spondyloarthritis (SpA) family and is relatively rare [7-9]. ReA is an oligoarthritis that is more common in males in the second and third decades of life and typically follows a distant extra-articular bacterial infection, usually enteric or urogenital, and is characterized by an acute sterile nonpurulent arthritis [7-10]. The most common cause of ReA is a sexually transmitted infection of *Chlamydia trachomatis,* which is asymptomatic in at least 70% of women and 50% of men at the time of diagnosis [11-13].

ReA usually manifests one to three weeks after the initial infection, but the mechanisms of etiopathogenesis remain unclear [12]. The arthritis is typically asymmetrical and predominantly affects the lower limb joints [12]. Contrary to septic arthritis, in ReA synovial fluid cultures are negative, suggesting that ReA is caused by an over-stimulated autoimmune response [9,12]. Tendinitis is a common finding of the disease and ReA can also present extra-articular manifestations, such as skin and mucocutaneous changes (e.g., hyperkeratotic skin; nail dystrophy), eye involvement (e.g., Conjunctivitis; uveitis) and in rare cases cardiac implications (e.g., conduction abnormalities; aortic regurgitation) [9,10,12].

The diagnosis is mainly clinical but complete laboratory tests, including detection of potential triggering pathogens, are usually performed to support the diagnosis and exclude other differential hypotheses, such as Gonococcal arthritis; Gout; Septic arthritis; other SpA diseases, and Rheumatoid arthritis [9,11-13]. There are still no diagnostic criteria for ReA. The American College of Rheumatology (ACR) proposed major and minor diagnostic criteria in 1999, but they have not been validated or widely adopted [9,11-13].

In elite professional level sports, there is a gap in the literature available. In this article, we outline the management and Return to Play (RTP) process after ReA diagnosis in an elite football player in the Portuguese first league.

## Case presentation

A 30-year-old male, professional football player, right-footed, with a relevant past medical history of bilateral grade IV chondromalacia in the Modified Outerbridge score [14], presented with bilateral knee pain and effusion two days after playing a game for his national team, with no identified trauma. Since he was abroad and based on his past medical history, he was prescribed at a distance with naproxen 1,000mg/day. After seven days with only mild resolution of the symptoms, he was asked to return to Portugal so we could make a definite diagnosis.

**Figure 1:**
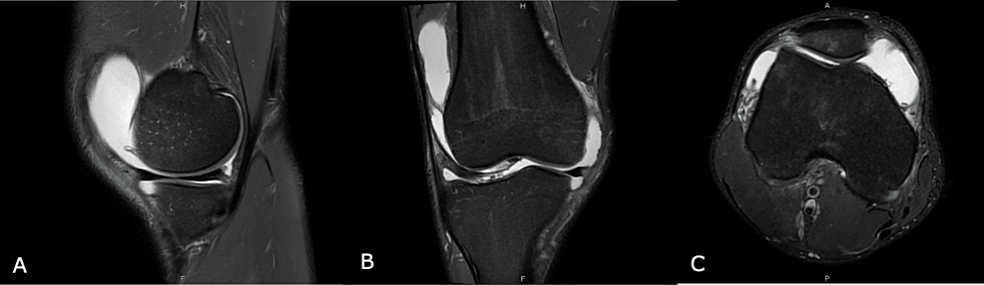
MRI of the right knee: T2-weighted sequence showing marked effusion with diffuse synovitis. (A) Sagittal plane. (B) Coronal plane. (C) Axial plane.

On examination, there was marked non-erythematous bilateral knee effusion with negative maneuvers for tendon, ligament or meniscal injury. He had 10 degrees deficit of flexion in the right knee and 7-degree deficit of flexion in the left knee, with no other restriction of range-of-motion (ROM) in any other joint. When asked he also reported inflammatory pain and early morning stiffness lasting 45 minutes, with no identifiable precipitating or relieving factors. An MRI scan was performed, showing marked effusion with diffuse synovitis (Figures 1A-1C). The next day, he presented with arthritis of the first metatharsalphalangeal joint in his right foot, and laboratory testing revealed inflammatory anemia Hb 12 g/dL (normal value: 13.2-16.6 g/dL); erythrocyte sedimentation rate (ESR) 48 mm (normal value: < 15mm); C-reactive protein (CRP) 1.37 mg/dL (normal value: < 0.50 mg/dL). 

Bilateral knee arthrocentesis was performed (right knee 40 mL; left knee 27 mL), results of which revealed 27,000 white blood cells/mm3, negative cultures and no crystals. *C. trachomatis* antibody IgG was positive and antinuclear antibodies 1/160. Serum test results were negative for HLA-B27, rheumatoid factor and anti-cyclic citrullinated peptide antibody. He later reported dysuria one month before, but no urethral discharge nor other genitourinary changes, no fever nor history of gastrointestinal, cardiac, ocular or dermatologic symptoms. Urine culture returned negative, including nucleic acid amplification test for *C. trachomatis* and *Neisseria gonorrhoeae*. Other possible sexually transmitted diseases were screened and noted to be negative.

After discussion with the Rheumatology consultant of the club, it was admitted a diagnosis of ReA after *C. trachomatis* infection. Started medication with esomeprazole 20 mg/day, doxycycline 200 mg/day, acemetacin 90 mg/day and after a request for therapeutic use exemption (TUE) to Portuguese Anti-Doping Agency, began prednisolone 15 mg/day.

In the first four weeks, he was restricted from physical activity and integrated a rehabilitation program with one hour of physiotherapy five times/week. Early rehabilitation main goals included reducing symptoms of acute inflammation and swelling manual therapy, ultrasound, LASER therapy, pain-free ROM exercises in all lower limb joints and compression cryotherapy in the affected joints. Laboratory tests were performed every week.

At week 4, after documentation of normal levels of ESR and CRP, doxycycline was suspended, the dose of acemetacin was reduced to 60mg/day and the athlete began a strengthening program respecting the isometric-concentric-eccentric order. At week 6, it was possible to introduce functional movement patterns with hydrotherapy and it was possible to intensify strength and conditioning in the gym. At week 10, it was possible to start jogging (5.5-6 km/h), with a progressive increase in speed. At week 11, he started the rehabilitation on the field with football specific work according to the *Control-Chaos Continuum *(CCC) [15,16].

The CCC is a framework proposed by Taberner et al., that guides the RTP process using retrospective player chronic workloads of global positioning systems (GPS) that helped us guiding phase progression, in a field where there is lack of evidence to support decisions [15]. Respecting the CCC, we were able to progress from high control to high chaos, setting running loads under gradually riskier conditions and at week 13 the athlete was able to return to training (RTT) after meeting all the clinical and functional criteria established by the Health and Performance department of the club, which included: absence of clinical symptoms; no pain or effusion in all joints; full ROM in all joints; normal levels of ESR and CRP; normal cardiac screening in the pre-competition medical assessment (including resting electrocardiogram, transthoracic echocardiogram and cardiac exercise stress test), free weight squat 1.5x body weight; quadriceps and hamstrings isokinetic dynamometric values ≥90% of the pre-injury values; *Psychological Readiness* and respect of CCC [15,16].

**Figure 2:**
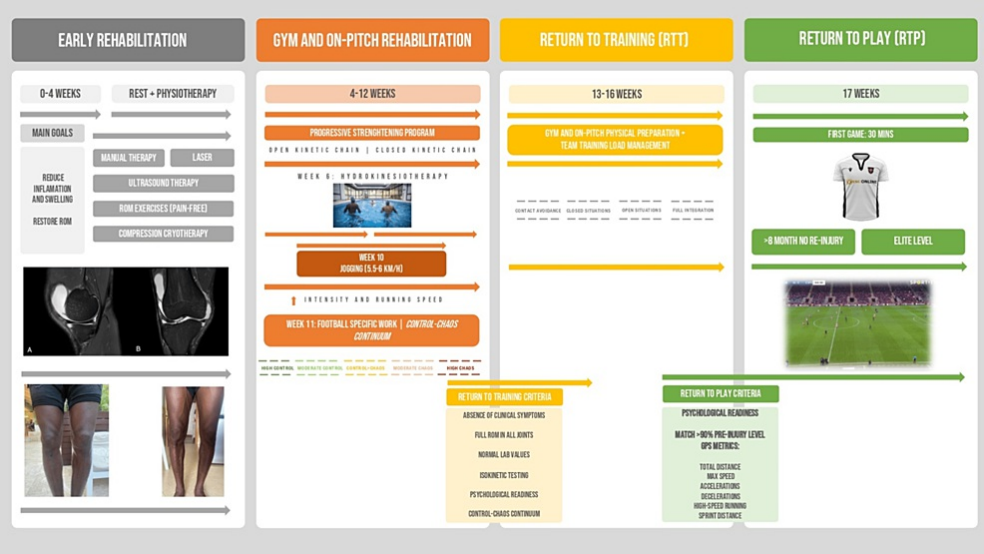
Return-to-play process infographic. Based on *Control-Chaos Continuum.*

The athlete was integrated in team training initially in closed and controlled situations, with progression to open situations. After four weeks of training with the team, the player matched >90% of pre-injury level GPS metrics and was physical and psychologically ready to RTP, 17 weeks after the diagnosis. Successful tapering of corticosteroids was clinically feasible in less than six months after diagnosis (Figure 2).

In the first game, the athlete played 30 minutes and eight months after RTP, the athlete had no ReA recurrence or injury and is competing at elite level. Intimate follow-up was made after RTP with daily assessment of the player signs and symptoms and laboratory testing was performed every month to document normal values of ESR and CRP.

## Discussion

Although most musculoskeletal complaints in Sports Medicine are traumatic or due to overuse, it is important to be cautious about other causes of joint pain and swelling, especially when symptoms do not improve with initial conservative therapy. Rheumatological causes may not be recognized, highlighting the importance of careful history taking and thorough physical examination, especially in athlete’s pain characterization to guide differential diagnosis [7,11,12,17].

ReA is a rare disease with a heterogeneous clinical presentation. Currently, there is no universally recommended treatment or well-defined guidelines, with management being largely supportive and tailored on a case-by-case basis [7,10,17].

First-line therapy usually includes relative rest with restriction of physical activity and physiotherapy with the main goals being reduction of inflammation and prevention of muscle wasting and, when symptoms improve, muscle strengthening and ROM restoration in the affected joints [10]. Non-steroidal anti-inflammatory drugs (NSAIDs) are usually effective, with no specific drug of choice, but are typically used in high doses [7,10,11,17]. As a second line and when arthritis pain is refractory to NSAIDs, systemic glucocorticoid therapy is frequently prescribed, but evidence supporting usage is limited [10,17]. The choice of dose and tapering regimen should aim for the lowest effective dose [7,17]. Antibiotic therapy is usually recommended for the treatment of concomitant urogenital chlamydial disease with a macrolide or tetracycline [7,10,11,17].

The course of ReA can be extremely variable ranging from a self-limiting process to a continuous, unremitting, and progressive disease [7]. Most patients have a self-limiting course of three to six months, but around 25% develop chronic SpA (i.e., longer than six months) of varying severity and require long-term immunomodulatory therapy such as disease-modifying antirheumatic drugs (DMARDs), like sulfasalazine and methotrexate, or biologic therapies such as anti-tumor necrosis factor (TNF) agents [7,11,12].

Different from other forms of SpA, the presence of HLA-B27 positivity in ReA is not considered useful for the diagnosis, but it is associated with the perpetuation of chronicity [7,11,12]. In addition, the elevation of ESR, lack of response to NSAIDs, and involvement of the hip joint are usually associated with poor prognosis [12]. Reactivation of the disease can occur in 25% to 50% of cases despite a cure and may signal a new infection or stress [12]. Furthermore, even if ReA is self-limited patients can develop secondary osteoarthritis (OA) [11].

In high-performance professional football, player absence due to injury/illness impacts not only the player’s integrity and physical performance but the club's financial outcomes [2,18]. Player readiness is of utmost importance in this high-demand environment, where the medical staff has the difficult task to enable athletes to achieve peak performance while safeguarding their health [19].

Rheumatic disease causes not only inflammation and damage to joints but can cause damage vital organs [20]. A high index of suspicion, early diagnosis, prompt treatment, and intimate follow-up are crucial for slowing disease progression with lower organ and joint damage, enabling the athlete to maintain the greatest physical function [5,11].

In elite football, evidence on the management of this condition is limited. To our knowledge, there is no published clinical case in professional football detailing the full RTP process following the diagnosis of ReA.

## Conclusions

With this clinical case, we illustrate the importance of an early etiopathogenic diagnosis. The sports medicine practitioner should be aware of rheumatic diseases to make an accurate diagnosis and prompt adequate treatment that can modify disease progression and prevent life-threatening conditions. In elite professional football, the fastest RTP is usually demanded, but we as medical professionals must make sure the athlete does it in the safest way possible. The player was able to RTP in the same season four months and one week after the diagnosis and is competing at elite level with no ReA recurrence or injury, eight months after RTP.
